# The Role of Neighborhood Environment in Promoting Risk Factors of Cardiovascular Disease among Young Adults: Data from Middle to High Income Population in an Asian Megacity

**DOI:** 10.1371/journal.pone.0124827

**Published:** 2015-05-06

**Authors:** Mohammad Ahraz Hussain, Sandal Noorani, Amna Khan, Hafsa Asad, Anam Rehan, Aamir Kazi, Mirza Zain Baig, Arish Noor, Amash Aqil, Nida Shahab Bham, Mohammad Ali Khan, Irfan Nazir Hassan, M. Masood Kadir

**Affiliations:** 1 Medical College, Aga Khan University, Stadium Road, P.O. Box 3500, Karachi, Pakistan; 2 Department of Community Health Sciences, Aga Khan University, Stadium Road, P.O. Box 3500, Karachi, Pakistan; University of Oxford, UNITED KINGDOM

## Abstract

**Background:**

Modifiable risk factors of cardiovascular diseases (CVD) have their triggers in the neighborhood environments of communities. Studying the environmental triggers for CVD risk factors is important to understand the situation in a broader perspective. Young adults are influenced the most by the environment profile around them hence it is important to study this subset of the population.

**Methods:**

This was a descriptive study conducted using the EPOCH research tool designed by the authors of the PURE study. The study population consisted of young adults aged 18-25 in two areas of Karachi. The study setting was busy shopping malls frequented by young adults in the particular community being studied.

**Results:**

Our total sample size was 120 individuals, who consented to be interviewed by our interviewers. Less than 50% of the population recognized some form of restriction regarding smoking in their communities. The largest contributor to tobacco advertising was actors smoking in movies and TV shows with 89% responses from both communities. Only 11.9% of the individuals disapproved of smoking cigarettes among men with wide acceptance of ‘sheesha’ across all age groups. Advertising for smoking and junk food was more frequent as compared to smoking cessation, healthy diet and exercise in both the areas. Unhealthy food items were more easily available in contrast to healthier options. The cost of healthy snack food options including vegetables and fruits was higher than sugary drinks and foods.

**Conclusion:**

This assessment showed that both communities were exposed to environments that promote risk factors for cardiovascular diseases.

## Introduction

World Health Organization reports that 36 million deaths occurred due to non communicable diseases [NCDs],(principally cardiovascular diseases, diabetes, cancer and chronic respiratory diseases) in 2008 which represents 63% of the worldwide mortality in that year. Cardiovascular diseases (CVD) were identified as the leading cause of death, amongst the NCDs (17 million deaths, or 48% of NCD deaths); with an additional 1.3 million deaths occurring due to diabetes mellitus. Over 80% of cardiovascular and diabetes deaths occurred in low- and middle-income countries. [1]

Acute MI occurs at ages 5 years younger in South Asian region compared to other countries.[2] ncds account for 59% of the total disease burden in Pakistan with cardiovascular disease representing the largest share followed by chronic respiratory diseases, cancer and diabetes reflecting a large number of lives lost due to ill-health, disability (DALYS), and early death. [1, 3]

A recent study focusing on the burden of NCDs in Karachi reported the prevalence of some risk factors for NCDs including tobacco use (45%), overweight (20%), abdominal obesity (53%), hypertension (18%), diabetes (8%) and pre-diabetes (40%) which are also ominously elevated. [4]

Awareness about CVD and its risk factors is an important requirement for the implementation of individual behavioral changes for its prevention. The general population has been shown to underestimate their individual risk for CVD risk factors.[5]

Studies conducted on the South Asian population also suggest a poor perception, awareness and degree of knowledge regarding CVD and its risk factors.[6–8]

Social, legislative, and physical domains and ecological settings within a community and the place of residence may potentially influence perception, awareness and health behaviors in relation to CVD risk factors (e.g. Smoking, diet, and physical activity) within populations. These domains include workplace smoking cessation programmes, smokefree bars and restaurants and opportunities for engaing in physical activity in environmental such as availability of built amenities for recereation. [9–15] Tobacco marketing and smoking in entertainment media has been shown to significantly impact increasing smoking behavior in younger age groups.[16–18] Associations between number of hours of TV watched and both the prevalence and incidence of obesity have been shown. [19]

Even though medical literature has addressed these triggers and has especially linked them to the prevalence of cardiovascular risk factors, there is scanty data concerning this from the developing world, especially South Asia. In view of the limited information available this report aims to evaluate the physical and social features of the built and neighborhood environments in Karachi, Pakistan for their role in promoting risk factors of cardiovascular disease including smoking, unhealthy diet and lack of physical activity.

## Methods

### Ethics statement

The study method, design and the instrument were approved by the Ethics Review Committee at the Aga Khan University, Karachi. Written informed consent was obtained from all the participants.

### Population setting and subjects

Karachi is Pakistan’s largest and most populous city. It is the country’s financial hub and the main seaport. It has a population of nearly 21 million, making it world’s third largest city by population.[20]

The study targeted young individuals, aged 18–25 years residing in two socio-economically similar communities in Karachi, namely Clifton-Defence and Tariq Road—PECHS both housing mostly middle to high income population. The study setting was one busy shopping mall and a busy 500m distance of a busy street in each area.

### Procedures

The methodology of this study has been adapted from that of the Prospective Urban Rural Epidemiology (PURE) study. Previously the impact of each risk factor on cardiovascular disease was evaluated separately. The EPOCH (Environmental Profile of Community Health)research tool created by the Population Health Research Institute (PHRI, Hamilton, Ontario, Canada) along with investigators at the London School of Hygiene and Tropical Medicine, and used in the PURE study, provides a way of analyzing all of these factors as a whole at a community level in different communities. [21]

The EPOCH tool, assesses the community from both the researcher’s and the respondent’s perspective. EPOCH 1 is an objective environmental audit tool in which trained researchers directly observe and systematically record physical aspects of the environment using a pro-forma, with standardized operational definitions. It has five sections, of which the initial part focuses on the environmental characteristics, including the transport, educational centers, factories, food markets and health care facilities. The second part consists of the “community observation walk”, the third part includes an assessment of the area’s tobacco stores. In section four, assessments regarding those community’s grocery stores are made. The last section takes into account several aspects of local restaurants, for example the importance they placed on healthy foods in the menus. In our study, while using EPOCH 1, the various aspects of the physical environment in a community were directly observed. A standardized protocol was followed for the observations made. This assessment requires 0.5km distance to be covered in the community while conducting the survey.

EPOCH 2 is an interviewer administered questionnaire that captures perceptions about the community from subjects living in that community. The four areas of interest in the EPOCH instrument are the tobacco environment, the physical activity environment, the food (including alcohol) environment, and the social and economic environment. These areas are evaluated using a series of 13 scales which are meant to measure different aspects of the community environment. Aspects of community awareness, attitudes, and social norms are assessed. These domains aimed to assess what the participants of the study observed within the confines of their community. In addition they aimed to assess their level of awareness regarding the existing laws, regulations and health programs. The last domain encompassed the aspect of opinions about those laws and behaviors.

This tool has successfully been used in China, India, Brazil, Canada and Colombia in urban and rural settings where researchers have obtained substantial information that can be generalized to the community. Out of these 5 countries two are neighboring Pakistan and hence it was a useful tool for our study.[22, 23]

#### Sample size for EPOCH 2

The total sample size of this study is 120, sixty individuals per community. This sample size was decided after reviewing the paper by DJ Corsi et al who have reported a reliability of >80% with a sample size of just 30 subjects per community for the EPOCH instrument. [24] Since a comparison of the two communities was not intended in this study the need to calculate a new sample size was not considered necessary. In order to increase the reliability of the data acquired the number recommended by the research tool’s authors was simply doubled for each community.

#### Subject Recruitment

The subject recruitment was done at shopping centers of the respective communities. The sampling methodology used was convenience sampling till the required number of subjects for each community were achieved hence the participation rate was 100%. All adults that visited the shopping mall were approached. The individuals were excluded if they were not residents of the local community. A screening question at the beginning of the question ensuring that the individuals filling out the questionnaire were in fact residents of that local community was included where residents were asked whether they lived within 500 meters of the shopping center. Individuals were also excluded if their ages were above 25 and if they were related to the medical field, eg. Medical and nursing students.

#### Pre-survey training and validation

The survey team comprised of fourth year medical students who were trained in the sampling protocol, interview process, and the method for completion of survey forms. The EPOCH (Environmental Profile of Community Health) manual was used to impart this knowledge to the team. This was done to ensure that all members provided a consistent explanation of questions to the participants. It also enabled the members to adopt an organized approach while conducting the community observation walk. Pretesting was done on ten non-medical university students, to estimate the amount of time taken to complete one questionnaire and to screen for problems in the translation and explanation of questions.

Data was entered using the SPSS version 19.0 database program. Frequencies and percentages, with 95% confidence intervals (CI) calculated for all the socio-demographic variables evaluated in this study.

## Results

### Epoch 1

Both the areas studied are residential plus commercial localities in Karachi. Tariq Road is situated in the heart of Karachi, surrounded by one of the oldest localities of the city, PECHS (Pakistan Employees Cooperative Housing Society). The locality consists of apartment buildings, town houses and bungalows. It is a famous shopping area in Karachi with malls, restaurants and shops located throughout its length, frequently crowded with shoppers throughout the year. Clifton is a similar neighborhood located in the south of Karachi. It is also a commercial centre, known for its availability of high-quality goods.

Both the areas, were quite similar to each other with regards to the regular network of transport for example, buses and shared taxis, facilities that were available for utilization by the residents of the community included supermarkets where food supplies could be bought, a general/convenience store, market stores such as vegetable store, bakery, butcher, fruit and tobacco shops.

EPOCH 1 includes an evaluation of the community with regards to its tobacco, nutrition, physical activity and food purchasing environments. These characteristics of the communities have been reported in Table 1.

**Table 1:** Availability of tobacco, fast food, fruits, vegetables and recreational parks in the two communities.

	Number of Observations
**Tobacco Street vendors/shops**	24
**Advertisements promoting cigarette products**	14
**Advertisements of sugary drinks and snack foods**	30
**Fast food restaurants**	13
**Fruit and vegetable vendors/shops**	18
**Advertisements promoting healthy diet**	3
**Recreation Parks**	1

In addition to these features it was noted that cigarettes and tobacco were openly displayed at the shops. They also had tobacco advertisements posted on the shops’ walls in addition to the display. A wide variety of cigarette packets were available at both locations including packs of 2, 10, 20 or 24 cigarettes. The packets at both locations displayed health warnings on the front, stating that smoking kills, causes mouth cancers and harms people around the smoker.

Two snack foods were studied as according to the requirements of the instrument. Both were produced in Pakistan, and the nutritional label provided information about items’ energy, total fat and saturated fat. Only one of the snack items that were evaluated provided additional information regarding the content of trans-fat. Non-commercial health promotions for a healthy diet were not found in either of the two communities.

Neither commercial nor non-commercial health promotions for physical activity were found in either community. Of the fast food places just one had highlighted healthy options, and included main dish salads, and vegetable dishes. All restaurants offered options to increase the portion size of client’s meal for a small price. The EPOCH 1 assessment also involves studying super stores/ supermarkets at both places. Supermarkets at both the locations had advertisements for sale for sugary drinks, while just one of them had sale advertisements for snack foods as well. None of the stores included fruits and vegetables in sales advertisements.

At both locations, a store was selected to evaluate their quality of display of fruits and vegetables. The first store/shop/vendor from the starting point of the walk was selected at both of these locations. The stores had the fruits and vegetables easily visible from outside; some fruits were specially packaged, wrapped or boxed for sale. Fruits on display at one of the areas however appeared to be damaged. None of the places had more than 3 kinds of vegetables that had been specially packaged, wrapped or boxed for sale.

Upon evaluating the public areas in the two residential areas for their environment in terms of promoting an environment for walking and physical activity it was noted that neither of them had flat, continuous sidewalks and street trees/street flower beds were present in just one of the two areas studied. One recreational park was identified near one of the areas only while. This recreational park near was under-constructed, and there were garbage heaps at two of its entrances. We did not expect to find some of the articles listed in both communities and it was rightly presumed. We did not find advertisements regarding alcoholic drinks nor health promotions for alcohol cessation. Alcohol specialty stores, pubs/bars were also not expected and neither were they found.

Food and tobacco purchasing environment at both locations was evaluated by recording the prices of the various items from general stores, street vendors, butchers, bakeries, and supermarkets. The prices are compared in Table 2. Price is an average of the prices recorded at both the areas. (sum of prices for each item divided by two)

**Table 2:** Food and Tobacco Purchasing Environment.

Item	Average prices of various food items (price in PKR, 1 USD = 105 PKR in January 2014)
**1 kg Apples**	125
**1 kg Oranges**	200
**1 kg Bananas**	110
**1 kg Carrots**	35
**1 kg Tomatoes**	45
**1 Medium sized cabbage**	30
**1 liter regular milk**	95
**1 liter low fat milk** (1%fat)	110
**1 loaf of white bread**	74
**1kg white rice**	150
**1kg chicken drumsticks with skin**	270
**1 egg**	6
**1 can/ bottle of cola**	45
**1 chocolate bar** (50g)	28
**Marlboro** (pack of 20 cigarettes)	130

### Epoch 2

The demographic characteristics of the respondents have been summarized in Table 3.

**Table 3:** Participants’ Demographics.

**Age** (Mean±SD)	21.0±2
**Education Status**(% of participants with mentioned education levels):	
• Senior/Higher secondary	34.5
• Trade school/College/University	65.5
**% Females**	61.1
**Smoking Status**	
• Current(%)	13.3
• Former (%)	12.0

As described in the methods section, the EPOCH 2 questionnaires consist of thirteen variables. Our results for these variables follow. The response rate of the individuals who were approached for this interview was 92%.

Smoking restrictions are evaluated using five questions in this instrument. These have been presented with the restriction preferences of the two communities in Table 4. The questions consist of items that evaluate the restriction of smoking in various places such as hospitals, public parks, bus stops etc. Less than 50% of the responses reported some form of restrictions regarding smoking in their locality. Interestingly, people would prefer stricter restrictions than that already in place in the community. This was found to be true for 51% of the responses.

**Table 4:** Smoking restrictions in neighborhood verses preferences regarding the restrictions.

	% ‘yes’ (95% CI upper, lower limits)
**Presence and severity of smoking Restrictions in public places**	
No restrictions	26.5 (22.8,30.2)
Some restrictions	48.5 (44.4,52.6)
Strict restrictions	20.0 (16.7,23.3)
**Preferences of the community regarding presence and severity of smoking restrictions in public places**	
No restrictions	8.2 (6.0, 10.4)
Some restrictions	3.1 (14.8, 21.0)
Strict restrictions	47.9 (43.8,51.9)

Tobacco advertising, (Table 5) is assessed by one question consisting of seven options that evaluates the participants’ observation of tobacco advertising in various areas of the community, for example advertisements on posters, billboards, and newspapers. Advertisements promoting tobacco are prevalent in general in both the communities, but the largest culprit are actors smoking in movies and TV shows as reported by 89% of the responses in both the communities. This was compared to the advertising of smoking cessation. We found that while the local media does promote smoking, more than half the respondents also acknowledged anti-smoking advertisements in their community.

**Table 5:** Tobacco advertising versus Promotion of Smoking Cessation.

	% ‘yes’ (95% CI upper, lower limits)
**Tobacco advertising in the community**	
Posters / billboards	49.6 (40.6,58.6)
Television / radio	48.7 (39.6,57.8)
Newspapers / magazines	47.0 (38.0,56.0)
Actors smoking in movies/ TV shows	89.0 (83.4,94.7)
**Promotion of Smoking Cessation**	
Posters / billboards	48.3 (39.2,57.3)
Television / radio	66.4 (57.9,74.9)
Newspapers / magazines	49.2 (40.2,58.2)

The general food environment is assessed using three variables, these include advertisements of junk food, fruits and vegetables and good dietary practices in various forms of media in the community. Like smoking, junk food advertisements are also prevalent in all media in the community. The variable, fresh fruit and vegetables advertising evaluated the participants’ observation of fresh fruit and vegetable advertisements in the community. Fresh fruits and vegetables and healthy dietary practices are notably less well advertised. The promotion of a healthy diet assessed the participants’ observation of campaigns that promote the importance of a healthy diet in the community. The results are illustrated in Table 6.

**Table 6:** Advertising for junk food vs advertising for fruits, vegetables and healthy diet.

	% yes (95% CI upper, lower limits)
**Presence of junk food advertising in various forms local media as reported by the individuals in the community**	
Posters	91.4 (86.3,96.5)
TV/Radio	91.4 (86.3,96.5)
Newspapers/magazine	86.2 (79.9, 92.5)
Sponsorship of sporting, music or other cultural events	69.8 (61.4,78.2)
Products such as umbrella, ashtrays, clothing	41.4 (32.4,50.4)
**Presence of fruit and vegetable advertising in various forms of local media as reported by the individuals in the community**	
Posters	37.9 (29.1,46.7)
TV/Radio	47.4 (38.3,56.5)
Newspapers/magazine	46.6 (37.5,55.7)
Sponsorship of sporting, music or other cultural events	25.9 (17.9,33.9)
**Advertisement of a healthy diet in general in various forms of local media as reported by the individuals in the community**	
Posters	33.6 (25.0,42.2)
TV/Radio	31.9 (23.4,40.4)
Newspapers/magazine	47.4 (38.3,56.5)

Knowledge of health effects of smoking and selected lifestyle changes in relation to heart disease in the community is displayed in Table 7. The questions consisted of eight items that evaluated the participants’ knowledge of the health effects of smoking and the diseases caused by smoking, for example the role of smoking in causing lung cancer, stroke, and heart disease. Majority of the participants were aware of smoking as a risk factor for heart disease, lung cancer and asthma. However, less than 40% could link smoking to arthritis and diabetes. The participants also displayed good knowledge with respect to most of the dietary practices and lifestyle interventions that protect against heart disease.

**Table 7:** Knowledge of smoking and lifestyle changes in relation to heart disease and other conditions.

	% Yes (95% CI upper, lower limits)
**Knowledge of health effects of smoking in both the communities**	
Heart disease in smokers	88.3 (82.6,94.1)
Arthritis in smokers	39.2 (30.5,47.9)
Stroke in smokers	62.5 (53.8,71.2)
Diabetes in smokers	34.5 (26.0,43.0)
Lung cancer in smokers	89.2 (83.7,94.8)
Lung cancer due to passive smoking	85.8 (79.6,92.1)
Asthma from passive smoking	72.5 (64.5,80.5)
Heart disease from passive smoking	55.8 (46.9,64.7)
**Knowledge of lifestyle interventions that protect against heart disease**	
More exercise	93.3 (88.8,97.8)
Eating more fruit	85.7 (79.4,92.0)
Eating more green vegetables	87.4 (81.4,93.4)
Eating more meat	16.0 (9.4,22.6)
Drinking more coffee	11.9 (6.1,17.7)
Eating more dairy products	35.3 (26.7,43.9)
Eating more fish	48.7 (39.7,57.7)
Smoking	28.6 (20.5,36.7)
Reducing fat in meals	77.8(70.3,85.3)
Reducing salt in meal	69.2(60.8,77.6)
Gaining weight	21.8 (14.1,29.5)

Social disapproval of smoking (Table 8) involved recording participants’ views about the perceptions of society regarding smoking. Majority of the respondents answered in the affirmative for the presence of social disapproval for smoking cigarettes among young adults and women, (54.2% and 68.9% respectively). However, only 11.9% of the individuals could report the same for men smoking cigarettes. For waterpipes the presence of disapproval was generally lower than other forms of inhaled tobacco.

**Table 8:** Social Disapproval of Smoking in the two communities.

	% Yes (95% CI upper, lower limits)	
**Social Disapproval of smoking**		
Smoking in children less than 15 years	77.5(70.0,85.0)	
Smoking in age groups 16–19 years	54.2(45.2,63.2)	
Women Smoking	68.9(60.6,77.2)	
Men smoking	11.9 (6.1,17.7)	
Smoking waterpipes (‘sheesha’)in age groups 16–19 years	19.2 (17.0,21.4)	
Smoking waterpipes in adults	20.5 (18.2,22.8)	
	% responses (95% CI upper, lower limit)	
	Common in neighborhood	Would not occur
Would adults in this community tell children, who are not their own children, to stop smoking?	27.8(19.7,35.9)	6 (1.7,10.3)
In your opinion, do people generally help others not related to them in this community?	35.3(26.7–43.9)	4.2(0.6,7.8)

Regarding awareness of tobacco and food legislation, most people were not fully aware of legislation governing smoking apart from mandatory warning on cigarette packages. 29.3% of the individuals were aware of any government/non-government support programs for individuals to stop smoking. 38.1% respondents were aware of laws that prohibit smoking in the youth. Participants’ awareness regarding food legislation in the community was observed using three questions. Less than 25% of the subjects were aware of any laws about food policy similar the case for smoking. The results are illustrated in Table 9.

**Table 9:** Community awareness regarding legislation and support programs.

	% Yes (95% CI upper, lower limits)
**Awareness of Tobacco legislation**	
Support programs that individuals can access to stop smoking	29.3(21.0,37.6)
Laws that ban smoking in public places	42.4 (33.5,51.3)
Laws that restrict tobacco advertising	42.4 (33.5,51.3)
Laws that mandate health warning on cigarette packs	66.9 (58.4,75.4)
Laws that prohibit smoking in youth	38.1 (29.3,46.9)
**Awareness of food legislation**	
Dietary guidelines or healthy food/habits	35.9(27.2,44.6)
Laws that mandate nutrient labeling on food/beverages	23.5(15.8,31.3)
Laws that subsidize or lower tax paid on fresh fruit and/or vegetables such that they are cheaper to buy	18.4 (11.3,25.5)

## Discussion

This assessment showed that middle to high income group communities in Karachi were exposed to environments that promote risk factors for cardiovascular diseases. It displays the imbalance in promotion and protection from risk factors of heart disease especially when comparing the numbers of tobacco shops and junk food restaurants with the number of fruit and vegetable vendors and recreational parks. (Table 1). Street foods contribute to the diet and also to the physical and social environments with respect to food in developing countries.[25] Neighborhood environment is associated with physical activity and overweight prevalence. [26] Accessibility and opportunities to facilities such as open space recreational parks and gyms plus the neighborhood surrounding’s aesthetic attributes are linked with physical activity.[27] The community-walk assessment done for the current report revealed disappointing characteristics of the communities’ environments with regards to physical activity. Communities do not seem to planned in a way that promotes more physical activity. We did not come across any data that assessed neighborhoods in South Asian cities for their health characteristics in relation to heart disease. Hence, topics such as walkability assessments of these cities need to be addressed in local public health research.

The prices of food items have a strong influence on individuals’ food choices. This study showed that the prices of high-carbohydrate, high-fat food groups (bread, full fat milk, white rice) is similar to high-protein, high-fiber, low-fat foods (chicken drumsticks, fruits, vegetables, and low-fat milk) in the local community.(Table 2) Lower prices of healthier food options would facilitate the general population in choosing them over the less healthier items available. Also, when it comes to making choices in snack foods and tobacco the prices of sugary drinks and foods and cigarettes are much lower than healthier alternatives like fruits and vegetables. Lower prices of tobacco and sugary foods in the community make it easier for the population to acquire them. This phenomenon has been recently reported in a study done on British young adults.[28]The situation is not different in rest of the world.[29–31] Increasing prices via taxes on cigarettes has been proven to help in discouraging its use with many additional public health benefits.[32]

It is encouraging to note where although both the communities were exposed to advertisements that promoted smoking, they were also aware of smoking cessation ads. However, actors in the local entertainment media such as movies and TV shows were found to have roles in which they were smoking. This was true according to 89% of the respondents of the two communities. Actors are often followed by their fans in many of their life aspects. Therefore, actors’ use of cigarettes and other forms of tobacco in the media may encourage their followers to do the same. Laws prohibiting the display of smoking in movies and TV shows are therefore required in Pakistan. Junk food advertisements were found to be much more frequent compared to promotion of fruits, vegetables and healthy diet in the communities’ neighborhoods. This was true across all forms of media. (Table 6) The paucity of advertising for healthy food options and healthy diet in the community could be due to the lack of financial resources of the health promotion programs versus the large and thriving junk food industry in Pakistan. A study conducted in the UK that explored variations in food and drink advertisement in 18 monthly women’s magazines over a 12 month period and found that only 3.9% of the total advertisements were for food and drinks. Of these, only 2% were for fruit and vegetables whereas 28% were for foods rich in fat/or sugar showing that most of these magazines feature a low proportion of healthier food items.[33]

Although local legislation exists in the country with regards to smoking and food, the communities were largely found to be unaware of any such laws. The legislation done in this regard includes the Bahawalpur State Juvenile Smoking Act, the West Pakistan Juvenile Smoking Ordinance 1959, Cigarettes (Printing of Warning) Ordinance 1979 and Prohibition of Smoking and Protection of Non- smokers Health Ordinance 2002. These laws prohibit smoking or use of tobacco in any form at public places and public vehicles, restrict tobacco advertisements, sales and storage of tobacco products near educational institutions. Laws also make it compulsory to print pictorial health warnings on cigarette packs and discourage ‘Designated Smoking Areas’.[34] The Pakistan Pure Food Laws was introduced in 1963 that addressed over 100 food items. It has instructions on using additives, storage, labeling and inspection of food items. Three additional laws regarding food safety in hotels and restaurants and in cantonment areas were formed in the later years.

The communities were gratefully aware of health effects of smoking and the lifestyle changes that may protect them from heart disease. The greater knowledge in these aspects may be due to them belonging to middle and high income groups. Adults in urban areas, generally show a good knowledge of health effects of smoking in their cohort. For example for 85% of the respondents were able to associate lung disease with smoking including cancer in South Africa. Our study population which is also urban shows a fair knowledge on adverse effects of smoking.[35]

However, advertisements, media and societal influences promoting smoking often defeat the benefit that can be achieved from this knowledge. In an assessment done on the Indian population it was found that even among knowledgeable men, 37.4% smoked and 33.2% had low fruit intake whereas among uneducated/without knowledge 55.6% smoked and 23.2% had low fruit intake. People belonging to low socioeconomic status had less knowledge.[36]

Shockingly enough majority of the respondents reported that their communities do not disapprove of men smoking especially considering the that male gender is a known non-modifiable risk factor for heart disease.(Table 8). More importantly, the social disapproval for adults and children (age groups <15 years) smoking waterpipes was not widely present. Waterpipes, sheesha’ exposes individuals to unfiltered tobacco along with exposure to carbon monoxide. The nicotine levels in individuals that smoke waterpipes have been reported to be equal to individuals that consume 10cigarettes/day. It has also been shown to increase nicotine/tobacco dependence with smokers having withdrawal symptoms, behavior changes in its absence and difficulty quitting. [37, 38]

We reviewed the available literature in medical publications and we were unable to find articles that have reported assessments on the social and physical environments of communities with respect to cardiovascular risk factors collectively. Two large multi-regional studies that have been launched in this context are the NESCAV study and the PURE study (the authors of which designed the research tool used in this study). [21, 39]

The strengths of our study include the research tool utilized for data collection which is well validated and has been an effective instrument in gathering information for the same purposes in neighboring countries such as India and China. Studies that have been previously carried out to assess the environment of individuals pertaining to cardiovascular risk factors have always focused on one risk factor and on people in higher age groups than 18–25 years. Our study has not only looked at the environmental profile collectively but also targeted the age group that is most amenable to environmental influences in adopting health behaviors in general.

All interviewers were well-versed in the instructions provided by the Epoch manual and were medical students who ensured that the individual responses were in line with the essence of the questions asked. All the interviewers belonged to the same age group as the respondents and this enabled the participants to be more comfortable and hopefully, honest in their responses.

The limitations in this study included the relatively small sample size. Although the sample size was sufficient for the reliability of the data recorded as per EPOCH instrument’s designers, its results cannot be generalized to the South Asian region. Since the study evaluated communities that belong to the middle socio economic strata, the results cannot be generalized to the low income groups. Because only urban communities were assessed the findings may not be applicable to the rural population in Pakistan. The prevalence of cardiovascular disease was not estimated in the study so we were unable to delineate the relationship between the environment of young adults that we assessed and the actual burden of cardiovascular disease in both areas. The subject of neighborhood environment in relation to community-level determinants of CVD risk factors has many more covariates that were not covered in this review such as literacy hence the recommendation that there is a need for more rigorous research protocols to investigate these to completion. Additionally since the method sampling used was convenience sampling there could be a chance for a selection-bias while interviewing the participants of this study.
